# Ingleby Lectures: Lecture VI

**Published:** 1891-05-16

**Authors:** 


### SURGERY.

### INGLEBY LECTURES.—Lecture VI.

#### Nævus.

Children are very prone to nævi, of which there are many varieties, and they may be found in any part of the body.

Pure nævi, *i.e.*, those consisting solely of capillaries of veins or of arteries, are exceptional, while mixed nævi are very common. These latter commence as small dots, slightly raised above the level of the skin, and vary much in their rate of growth.

The proper time to treat nævi is at their earliest appearance, and then you easily cure them by making the point of a needle red-hot and puncturing the spot with it deeply in one or more places. They rarely are seen in the hospitals at this early stage, and at a later stage many methods have been devised for their cure, as pressure, needling, subcutaneous sections, vaccination, blistering, the application of caustics, the injection of coagulating fluids, electrolysis, ligature, the actual cautery and excision.

Of these methods the best, Mr. Lloyd thinks, are *excision* and the *actual cautery*. The injection of perchloride of iron should never be practised; it is very dangerous. Mr. Lloyd quoted a fatal case he had seen. Excision is inapplicable to many nævi of the face, especially those of the lips, eyelids, and nose; the nævi of the scalp, which are large or situated over open sutures or fontanelles; the nævi of the tongue or parotid region. In such cases try ignipuncture, electrolysis, or pressure.

#### Foreign Bodies in the Air-Passages.

A bead in the *nose* is frequently overlooked. It cannot be seen through the anterior nares, and until ozæna supervenes its presence is not suspected. Ozæna in the child, especially if it only attacks one nostril, should always make you suspect a foreign body. To determine its presence it is best to give an anæsthetic, take a probe, bend its flat end so as to somewhat resemble an aneurism needle, and pass this into its curve downwards towards the roof of the nose, as far back as you can, then depress the point, and drag along the floor of the nostril as you withdraw the needle.

The *mouth* is the most common receptacle for foreign bodies, which may pass from there into the pharynx and œsophagus or into the larynx and trachea; the former is the rule, and then diagnosis and treatment is not generally difficult.

In the *pharynx*. It is best if you suspect a foreign body in the pharynx to inspect with a good light; then pass the finger along the dorsum of the tongue into the glosso-epiglottidean pouch; next sweep it over the fauces and press it between the pillars, and then slip it into the pharynx. If a foreign body can be felt it can easily be removed.

In the *œsophagus*. Pain and dysphagia are the indications of a foreign body in the œsophagus. If impacted, you can push on into the stomach all substances that are digestible; of the indigestible substances, if they are smooth, you can still do the same; if rough or pointed, they must be removed by the mouth by means of the probang, coin catcher, forceps, or by external incision if necessary.

In the *larnyx* and *trachea*. The presence of a foreign body in the air-passages is a condition fraught with danger, but its recognition is rarely difficult, and its treatment is decisive. The nature of such cases is often, however, overlooked—indeed, sometimes revealed only on the post-mortem table. A child who is suspected of having swallowed something and is subject to attacks of paroxysmal dyspnœa and paroxysmal cough should be assumed to have that something in the air-passages, until the contrary can be demonstrated beyond doubt. If the paroxysms keep recurring, and in between them there is an interval of complete quiet, no symptoms of any kind, your diagnosis is almost absolute.

Of course, tracheotomy is the only remedy, and if in doubt, you give your patient the benefit of the doubt by opening the trachea and not leaving him to chance. Mr. Lloyd quoted a case when he removed a locust stone 167 days after it had been swallowed, from the larynx of a girl aged five years, being guided in his diagnosis by the frequent paroxysmal attacks.

#### Tracheotomy.

A few practical hints may here be given with regard to this operation. Always give chloroform; steady the trachea with a finger and thumb; make your skin incision long enough, and in the middle line; separate the laryngeal muscles thoroughly with the handle of the scalpel; remember you now come on a stout layer of cervical fascia, in which are found the chief veins; divide this; be sure you distinctly see the rings of the trachea; hook up the windpipe with a sharp hook before incising it, and use a tracheal dilator to facilitate the introduction of the tracheotomy tube. If after opening the trachea the foreign body cannot be seen, invert the patient and slap the back; never do this before you have opened the trachea. If you then cannot see or feel the substance, put in a large trachea tube and wait for the substance to be coughed out.

#### Rickets.

This disease is very common amongst children; it is often overlooked in its earliest stages before bone deformity is present. The administration of an increased quantity of fatty food is very important in its therapeutic treatment.

*Surgical Treatment of Bow-legs and Knock-knees.*—These, in the majority of cases, if taken in time, can be cured without the aid of cumbersome apparatus or of surgical operation. To do this it is necessary that the parents possess patience, intelligence, and faith in the treatment, and that the child can be properly ruled. The patient must be taken off its legs altogether during the whole period of treatment. If the disease is bilateral, as it mostly is, firmly bandage the legs together from the toes to the buttocks with a soft pad between the knees in a case of genu valgum—between the internal malleoli in bow-legs. Three or four months of this treatment may be necessary in bad cases. If from various reasons mothers will not efficiently carry out this treatment, the limbs must be put straight by surgical means. Mr. Lloyd makes use of one of the following methods.

I. *Simple Fracture*, by means of some kind of osteoclast, or by the following method, viz., breaking the bone by forcibly bending it against the edge of the table or across a firmly padded bar.

It is easy to break a bone just at the spot required thus, and with very good results. The after treatment is that of a simple fracture. In thus breaking a bone, it is important that it should be held at its extremities and fulcrumed across the padded bar at the exact spot where you wish it to break.

II. *Osteotomy.*—If the bone cannot be broken thus, one must proceed to "osteotomy."

In *Genu valgum*, Mr. Lloyd prefers Macewen's supra-condyloid incision.

In *Bow-legs*, a simple osteotomy is only necessary in simple cases; when the curve is pronounced, excision of a wedge is necessary.

Mr. Lloyd quoted the cases of 125 osteotomies which he had performed on children under twelve years. He had only had one death—that was due to secondary shock. Beyond this he had not had complications of any kind occur, and he thought the record was one of which antiseptic surgery might be proud.

